# Emotional and behavioral problems in children and adolescents with hearing impairment

**DOI:** 10.1186/s12887-025-05696-4

**Published:** 2025-05-09

**Authors:** Dalia Fahim Mohammed Fahim, Sara Ibrahim Sayed, Marwa Ibrahem Abdelrazic, Ibtehal Saad Abuelela

**Affiliations:** 1 Audiovestibular Medicine, ENT Department, Minia University, El-Minia, Egypt; 2 Department of Public Health and Preventive Medicine, Faculty of Medicine, Minia University, El- Minia, Egypt; 3 Department of Pediatrics, Faculty of Medicine, Minia University, El-Minia, Egypt

**Keywords:** Hearing impairments, Children, Emotional and behavioral disturbance, Conduc

## Abstract

**Background:**

Hearing impairment (HI) is a prevalent problem. The majority of cases have HI during the neonatal period, while the still cases have incidence up to adolescents. Hearing amplification is a promising modality to restore hearing with promising effects on speech and communication. The objective of this study is to assess the effect of being a child with HI on emotions and behaviors despite using a hearing amplification device early in life.

**Patients and methods:**

The study is a cross-sectional descriptive study including 127 children aged from 4 to 17 years diagnosed with hearing loss; 71 were with hearing aids and 56 with cochlear implants recruited from the audio-vestibular unit, ENT department and referred to the child psychiatry clinic, department of pediatrics, Minia university, Egypt. The questionnaire used in this study is the parent-rated strength difficulty questionnaire (SDQ), which consists of 5 main components, namely “emotional problems,” “conduct problems,” “hyperactivity/Inattention problem,” “peer relationship problems,” and “prosocial behavior”.

**Results:**

On stratifying the patients according to the degree of sensorineural hearing loss, 66.6% of children with lesser degrees of hearing loss showed significant conduct behaviors. While on stratifying the patients according to the age groups; 86% of primary school children experienced significant peer relationship problems (*p* = 0.03), while 66.6% of children in the late childhood period had significant emotional disturbances (*p* = 0.023). On regrouping of the patients according to the type of amplification (hearing aids vs. cochlear implants) and side of amplification (right, left, or bilateral), no significant differences in emotional and behavioral disturbances were observed in any group.

**Conclusion:**

Despite receiving early amplification, children with hearing loss still develop emotional and behavioral problems, with children who had a milder degree of hearing loss developing significant conduct behaviors. Additionally, those children developed significant peer relationship problems at school entry age, problems that older children can overcome, but with significant internalizing symptoms and emotional disturbances. These problems need more community orientation and psychological support to the child and their family, particularly during the transition to school. Furthermore, early screening and intervention for emotional disturbances in adolescents are essential to ensure timely management and support.

**Clinical trial number:**

Not applicable.

## Introduction

Communication is essential for the development of the child because it facilitates interpersonal interactions and it helps control behavior [1, 2]. Communication requires normal hearing and normal language development as auditory comprehension precedes the normal development and acquisition of language and speech [3, 4]. Hearing impairment (HI) occurs when there is reduced sensitivity to sounds normally perceived [5]. Children with HI may experience many difficulties in their development because they lack completely developed speaking and listening skills and are unable to talk functionally since they cannot hear the language spoken around them, which can limit communication [4, 6]. Language is a critical factor in the development of an individual, as it not only facilitates social exchange but also assists in the internalization of social norms and the development of behavioral control [7]. Hence, delayed language development could serve as the fundamental root of undesirable habits [8, 9].

Hearing loss is the most common sensory deficit and one of the most common congenital abnormalities [10]. Overall, the prevalence of newborn persistent sensorineural hearing loss (SNHL) is 0.2%, with a range of 0.1–0.6% [11]. Children with HI since birth often have developmental gap, because of sustained auditory deprivation since birth. So, early identification of HI and treatment, including hearing amplification within 6 months produces a favorable impact and superior outcomes for development, as the duration of hearing loss has significantly decreased [12]. It is possible to detect HI in infants through hearing screening programs in the first few days following delivery [13]. When the HI is detected later, hearing impaired children do not encounter normal auditory surroundings and can suffer from a variety of daily life challenges [14–16].

The use of cochlear implants (CI) and hearing aids (HA) helps patients who are hearing impaired to improve their auditory perception. This, in turn, enables them to participate more actively in the community by speaking, which is a common form of interpersonal communication. The majority of published studies measured the direct effects of amplification, including sound and speech perception and production [17, 18]. Long-term effects of children’s functioning in daily situations, such as their communication skills, interactions with the social environment at home and at school, and their desires and needs, will become easier to report over time [19].

HI not only has an effect on the persons themselves, but also on their parents and the environment in which they live; hence, it is also possible to take into consideration the social effects of HI. Consequently, it would be incorrect to view this situation as a disability alone [20]. In comparison to their healthy peers, children with SNHL who were left unassisted exhibited a higher incidence of behavioral problems, including aggressiveness, conduct problems, inattention, hyperactivity problems, and mood disorders [21, 22]. Children with HI are more likely than their peers with normal hearing to experience internalizing (such as anxiety and sadness) and externalizing (such as hyperactivity and behavior issues) disorders [23].

Children with HI not only benefit from CI and HA in terms of hearing restoration, but they also showed noticeable suppression of these behavioral problems [24, 25]. A variety of factors can influence their capacity to develop social skills and communicate effectively in group settings. Based on these factors, we can predict the psychological and social effects of hearing devices [26]. These factors include the type of hearing device [6], the degree of hearing loss [27], the child’s academic achievement, the ability to perceive speech in both quiet and noisy environments [28], the employment status of the mother, and the number of children in the family [29].

The objective of this study was to describe the relationship between hearing loss, intervention timing, type of intervention and behavioral outcomes, which includes; “emotional problems,” “conduct problems,” “hyperactivity/Inattention problem,” “peer relationship problems,” “total difficulties score” and “prosocial behavior”.

### Patients and methods

#### Study design

The study is a cross-sectional descriptive study including 127 children aged from 4 to 17 years diagnosed with hearing loss, 71 were with HA and 56 with CI recruited from audio-vestibular unit, ENT department and referred to child psychiatry clinic, department of pediatrics, Minia university, Egypt. The sample size was calculated using the formula of Daniel et al. n = Z^2^*P* (1 − P) /d^2^] [30], where; n = Sample size, Z = Z statistic for a level of confidence (1.96 for 95% confidence level), P = Expected prevalence was found to be 9% and, d = Precision. Then, the sample size was determined as n= (1.96)^2^* 0.09* (1–0.09)/ (0.05)^2^ = 127 [31].

***The inclusion criteria*** were: (1) Children diagnosed with hearing loss, (2) Aged from 4 to 17 years, (3) Using HA and/or CI. ***The exclusion criteria***were:1) having major neuropsychiatric problems as autism and epilepsy, and 2) any patient refused to be enrolled in the study.

### Ethical consideration

The Institutional Review Board of Minia University approved the study with NO: 817/6/2023. Before initiation of the study, the aims and design of the study were explained for the guardians of those children, and only agreed parents were enrolled in the study. Written consent was taken from the children’s guardians, and verbal consent was taken from the children themselves.

### Methods

The Guardians of the children were met by a pediatrician to answer a structured questionnaire about some demographic data concerning their children’s age of diagnosis of hearing loss, time of intervention (either by HA or CI), type of school, and delay in school achievement. Also, questions related to some risk factors like prenatal, perinatal, neonatal, and postnatal history, including complication of gestations, type of delivery, birth complication, jaundice, NICU admission, fever, and trauma, as well as family history. The children were met and assessed to exclude any associated overt mental illness. The children with their guardians were then referred to an expert psychologist to apply the strength and difficulty questionnaire (SDQ). Each child has a hearing assessment and a psychometric assessment.

### Hearing assessment

Based on the patient’s age and reliability, either conditioned play audiometry or standard audiometry is used to evaluate hearing sensitivity. The degree of hearing loss (mild, moderate, moderately severe, severe, profound) was calculated for the best hearing ear in the HA patients and the worse ear in the CI patients based on the average air conduction thresholds at 0.5,1,2 and 4 kHz. The type of hearing loss (sensorineural, conductive, and mixed) was determined in patients based on the average bone conduction thresholds at 0.5,1,2 and 4 kHz. Speech discrimination score (SD score) used to assess how well an individual can understand speech. The score (excellent, good, fair, poor, and very poor) was determined [32].

### Psychometric assessment using strength and difficulty questionnaire (SDQ)

The SDQ is a concise behavioral screening questionnaire that is designed for children and adolescents. It exists in several versions targeted at parents, researchers, clinicians, teachers, and children/adolescents. Each version includes between one and three of the following components: “emotional problems,” “conduct problems,” “hyperactivity/Inattention problem,” “peer relationship problems,” “total difficulties score,” and “prosocial behavior” [33].We used the pre-prepared, validated parent-version SDQ for children aged 4 to 17 in Arabic. The questionnaires consist of 25 items that parents or teachers of children aged 4 to 17 must complete. It has been validated for identifying psychosocial disorders, children’s abilities, and the effects of psychosocial difficulties on everyday life activities in both medical diagnosis and scientific research [34–36].

The questionnaire used in this study is parent rated SDQ. It consists of 5 main scales namely (1) emotional problems (5 items), (2) conduct problems (5 items), (3) hyperactivity/inattention problems (5 items), (4) peer relationship problems (5 items) and (5) prosocial symptoms (5 items). Total score difficulty is generated by adding 1 to 4 scales (based on 20 items).

***The emotional problem scale questions*** are mainly about somatic, worries, being unhappy, clingy and afraid, ***the conduct problem scale questions*** cover the item about tantrum, obedient, fights, lies and steals, ***the Inattention/Hyperactivity problem scale questions*** cover the items of being restless, fidgety, distracted, reflect and, attend, ***the peer relationship problem scale questions*** mainly cover items of being loner, friend, popular, bullied and old best and finally ***the prosocial behavior scales questions*** cover the main items of considerate, sharing, caring, kind, and helpout. Each question will be answered on the frequency Likert scale (always, sometimes, and never), which is scored as (2, 1, 0), respectively. However, questions 7, 11, 14, 21, 25 of prosocial behavior scale were reversely coded (0 = 2, 1 = 1, 2 = 0).

The total score of the whole scale and the sum of the answers of the 5 questions of each scale will be arranged differently according to each scale and will be represented through items of average, raised, high, and very high. The sum of the scores of the first 4 subscales (“emotional problems,” “conduct problems,” “hyperactivity/Inattention problem,” “peer relationship problems,” and “total difficulties score”) provides a behavioral and emotional problems score ranging from 0 to 40. Higher scores coincide with more behavioral difficulties. However, higher scores on the 5th subscale (prosocial behavior) reflect optimized behavior in society [37]. Except for the prosocial scale, this will be represented in a reverse manner: average, slightly low, low, and very low. The Arabic version of the SDQ was validated by Alyahri et al. in 2006 [38].

Each of these scales is scored from 0 to 10 and can be classed depending on how the score compares with population standards based on original validation work in the UK as follows (80% ‘close to average’, 10% ‘slightly raised, 5% ‘high’, and 5% ‘very high’ for all scales except prosocial, which is 80% ‘close to average’, 10% ‘slightly lowered’, 5% ‘low’ and 5% ‘very low’ [39].

### Statistical analysis

IBM SPSS version 25 (IBM Corp., 2017) was used for statistical analysis. IBM SPSS Statistics for Windows, Version 25.0. Armonk, NY: IBM Corporation. Data normality was assessed via the Kolmogov-Smirnov test; as a result, the Kruskal Wallis test was used for non-parametric quantitative data between three groups, and the Mann Whitney test was used for quantitative data between two groups. Fisher Exact test/Chi square test was used to compare between two or more groups as regard qualitative data. Multiple linear regression analysis for determining factors affecting scales of SDQ. Adjustment was made for age, age at diagnosis, sex, degree of hearing loss, school achievement, and degree of discrimination, the delay time of intervention, laterality, and aiding device. A p-value less than 0.05 was considered statistically significant.

## Results

A total of 127 children were included in this study. Seventy-one of these children were HA users, and 56 were CI users. The median of their ages was 8 years (5–11). There were 55.9% boys and 44.1% girls, and about 90% of children in the HA group had SNHL. About half of these patients (46.5%) had a moderately severe degree of hearing loss and a zero to poor SD score. The median age of their diagnosis was 3 years (2–4) with 0.8 year (0.25–1) time delay to interference. More than half of these patients (63.4%) use bilateral HA (binaural). About 77.5% of these patients attend school (almost ordinary school), and about 80% of them have normal school achievement. In Comparison to the children in the CI group, all patients had SNHL, mostly of a profound degree, and zero to poor speech discrimination scores. Their median age of diagnosis was 1.5 years (1- 2.5) and 2 years (1–3) time delay to interference. About 80.4% use their CI in the right side (monaural HA). Half of these patients (50%) attend school (an ordinary school), and more than half of them have normal school achievement. There was a statistically significant difference between the two groups, as shown in Table 1.

**Table 1:** Demographic characteristics and audiological examination of the studied group

Variable	All patients *N* = 127	Hearing aid group *N* = 71	Cochlear implant group *N* = 56	*P* value
Age (years)				
Median (IQ)	8 (5–11)	9 (6–13)	6.5 (5–9)	0.002*
**Sex**				
Boys Girls	71 (55.9%) 56 (44.1%)	43 (60.6%) 28 (39.4%)	28 (50%) 28 (50%)	0.281
**Type of hearing loss**				
Conductive SNHL Mixed	1 (0.8%) 120 (94.5%) 6 (4.7%)	1 (1.4%) 64 (90.1%) 6 (8.5%)	0 (0%) 56 (100%) 0 (0%)	0.034*
**Degree of hearing loss**				
Moderately severe Severe Profound	33 (26%) 21 (16.5%) 73 (57.5%)	33 (46.5%) 20 (28.2%) 18 (25.4%)	0 (0%) 1 (1.8%) 55 (98.2%)	0.001*
**Degree of discrimination**				
Zero to poor Fair Good to excellent	88 (69.3%) 11 (8.7%) 28 (22%)	33 (46.5%) 10 (14.1%) 28 (39.4%)	55 (98.2%) 1 (1.8%) 0 (0%)	0.001*
**Age of diagnosis**				
Median (IQ)	2 (1–3)	3 (2–4)	1.5 (1–2.5)	0.001*
**Time delay to interference**				
Median (IQ)	1 (0.5–2)	0.8 (0.25–1)	2 (1–3)	0.001*
**Age of hearing device fitting**				
Median (IQ)	4 (3–5)	4 (3–6)	4 (3–4.5)	0.905
**Laterality**				
Bilateral Right Left	45 (35.4%) 62 (48.8%) 20 (15.7%)	45 (63.4%) 17 (23.9%) 9 (12.7%)	0 (0%) 45 (80.4%) 11 (19.6%)	0.001*
**Attendance at school**				
Not yet Going to school	44 (34.6%) 83 (65.4%)	16 (22.5%) 55 (77.5%)	28 (50%) 28 (50%)	0.001*
**Type of school**	*N* = 83	*N* = 55	*N* = 28	
Deaf and Mute Integrated Ordinary school	2 (2.4%) 5 (6%) 76 (91.6%)	1 (1.8%) 1 (1.8%) 53 (96.4%)	1 (3.6%) 4 (14.3%) 23 (82.1%)	0.066
**School achievement**	*N* = 83	*N* = 55	*N* = 28	
Normal Delayed	59 (71.1%) 24 (28.9%)	44 (80%) 11 (20%)	15 (53.6%) 13 (46.4%)	0.012*

- Mann-Whitney U test used to compare non-parametric quantitative data between two groups

- Chi square test / Fisher Exact test was used to compare qualitative data between two groups

*: Significant difference (p value ≤ 0.05)

-IQ (Interquartile range)

The severity of hearing loss in relation to the socio-demographic data, some risk factors, and audiological examination illustrated in Table 2. Most of the children in the study had SNHL. Those with profound hearing loss had more affected speech discrimination than those with severe and moderately severe hearing loss. About 97% of profound hearing loss patients had zero to poor degrees of discrimination in comparison to 15.2% and 57.1% for moderately severe and severe hearing loss patients, respectively. Patients with profound hearing loss diagnosed earlier and consumed more time for interference than those with severe or moderately severe hearing loss, however, there was no significant difference between different degrees of hearing loss and the age of HA fitting. There was a statistically significant difference between the three groups as shown in Table 2. About 67% of profound hearing loss patients used hearing amplification devices, either HA or CI at the right side (monaural) compared to 57.6.% of moderately severe and 61.9% of severe hearing loss use binaural HA, this of significant difference. Most of the studied groups went to ordinary school with almost normal school achievements in those with moderately severe and severe hearing loss compared to more delayed in school achievement in those with profound hearing loss. There were no significant relations between different degrees of hearing loss, mode of delivery, post-natal illness, management of jaundice, family history, and consanguinity.

**Table 2:** Degree of hearing loss in relation to the sociodemographic data, some risk factors, and audiological examination of the studied group

Variable	Moderately severe *N* = 33	Severe *N* = 21	Profound *N* = 73	*P* value
Age (years)				
Median (IQ)	9 (6.5–13)	8.5 (6–11)	7 (5–10.5)	0.002*
**Sex**	22 (66.7%)	13 (61.9%)	36 (49.3%)	
Boys Girls	11 (33.3%)	8 (38.1%)	37 (50.7%)	0.208
**Type of hearing loss**				
Conductive SNHL Mixed	0 (0%) 31 (93.9%) 2 (6.1%)	1 (4.8%) 17 (81%) 3 (14.3%)	0 (0%) 72 (98.6%) 1 (1.4%)	0.014*
**Degree of discrimination**				
Zero to poor Fair Good to excellent	5 (15.2%) 7 (21.2%) 21 (63.6%)	12 (57.1%) # 3 (14.3%) 6 (28.6%)	71 (97.3%) $ 1 (1.4%) 1 (1.4%)	0.001*
**Age of diagnosis**				
Median (IQ)	3 (2–5)	3 (2.25–4)	1.5 (1–2.5) $	0.001*
**Time delay to interference**				
Median (IQ)	1 (0.5–2.25)	0.5 (0–1)	1.5 (0.52–2.08)	0.001*
**Age of hearing device fitting**				
Median (IQ)	4.95 (3–7)	4 (2.75–5)	3.5 (3–4.5) $	0.905
**Laterality**				
Bilateral Right Left	19 (57.6%) 10 (30.3%) 4 (12.1%)	13 (61.9%) 3 (14.3%) 5 (23.8%)	13 (17.8%) $ 49 (67.1%) 11 (15.1%)	0.001*
**Attendance at school**				
Not yet Going to school	9 (27.3%) 24 (72.7%)	4 (19%) 17 (81%)	31 (42.5%) 42 (57.5%)	0.081
**Type of school**	*N* = 24	*N* = 17	*N* = 42	
Deaf and Mute Integrated Ordinary school	0 (0%) 1 (4.2%) 23 (95.8%)	0 (0%) 1 (5.9%) 16 (94.1%)	2 (4.8%) 3 (7.1%) 37 (88.1%)	0.682
**School achievement**	*N* = 24	*N* = 17	*N* = 42	
Normal Delayed	23 (95.8%) 1 (4.2%)	11 (64.7%) # 6 (35.3%)	25 (59.5%) $ 17 (40.5%)	0.006*
**Mode of delivery**				
CS SVD	20 (60.6%) 13 (39.4%)	8 (38.1%) 13 (61.9%)	38 (52.1%) 35 (47.9%)	0.272
**Postnatal illness**				
Normal Jaundice Others	20 (60.6%) 9 (27.3%) 4 (12.1%)	13 (61.9%) 6 (28.6%) 2 (9.5%)	43 (58.9%) 26 (35.6%) 4 (5.5%)	0.709
**Management of jaundice**	*N* = 9	*N* = 6	*N* = 26	
No Photo Incubated	6 (66.7%) 1 (11.1%) 2 (22.2%)	3 (50%) 0 (0%) 3 (50%)	12 (46.2%) 8 (30.8%) 6 (23.1%)	0.322
**Family history**				
Negative Positive	17 (51.5%) 16 (48.5%)	13 (61.9%) 8 (38.1%)	48 (65.8%) 25 (34.2%)	0.378
**Consanguinity**				
Negative Positive	13 (39.4%) 20 (60.6%)	6 (28.6%) 15 (71.4%)	22 (30.1%) 51 (69.9%)	0.592

- Kruskal Wallis test for quantitative data between three groups

- Mann-Whitney U test used to compare non-parametric quantitative data between two groups

- Chi square test / Fisher Exact test was used to compare qualitative data between two groups

*: Significant difference (p value ≤ 0.05)

#: Significant difference between moderately sever and sever

$: Significant difference between moderately sever and profound

-IQ (Interquartile range)

The relation of SDQ to the degree of hearing loss is shown in Table 3. On stratifying the patients into 3 groups according to the severity of hearing loss- moderately severe, severe, and profound hearing loss- our results revealed no significant differences in the SDQ scale results between the 3 groups except for conduct behavior, which is more evident in children with moderately severe hearing loss. More than half of the children with moderately severe hearing loss group (60.6%) had conduct problems to different degrees. About a third (30.3%) of those children were in the very high score (i.e., very high conduct or behavioral problem). In comparison, children with severe and profound HL groups had conduct problems in 52.4% and 48%, respectively, with different degrees [only 9.5% and 5.5% of those children in the very high score respectively]. Near 40% of them were in the average score (no conduct or behavioral problem), in comparison to about half of the children of the severe and profound HL groups were in the average scores and this of significant importance.

**Table 3:** Relation between SDQ and severity of hearing loss

SDQ Scale	Moderately severe *N* = 33	Severe *N* = 21	Profound *N* = 73	*P* value
Emotion scale				
Average Raised High Very high	16 (48.5%) 7 (21.2%) 3 (9.1%) 7 (21.2%)	7 (33.3%) 3 (14.3%) 4 (19%) 7 (33.3%)	32 (43.8%) 12 (16.4%) 12 (16.4%) 17 (23.3%)	0.798
**Conduct scale**				
Average Raised High Very high	13 (39.4%) 1 (3%) 9 (27.3%) 10 (30.3%)	10 (47.6%) # 5 (23.8%) 4 (19.1%) 2 (9.5%)	38 (52%) 20 (27.4%) 11 (15.1%) 4 (5.5%)	0.002*
**Hyperactivity scale**				
Average Raised High Very high	19 (57.6%) 3 (9.1%) 5 (15.2%) 6 (18.2%)	16 (76.2%) 2 (9.5%) 1 (4.8%) 2 (9.5%)	51 (69.9%) 5 (6.8%) 10 (13.7%) 7 (9.6%)	0.703
**Peer relationship scale**				
Average Raised High Very high	8 (24.2%) 8 (24.2%) 10 (30.3%) 7 (21.2%)	7 (33.3%) 4 (19%) 6 (28.6%) 4 (19%)	23 (31.5%) 26 (35.6%) 8 (11%) 16 (21.9%)	0.218
**Total scale**				
Average Raised High Very high	14 (42.4%) 6 (18.2%) 1 (3%) 12 (36.4%)	9 (42.9%) 3 (14.3%) 4 (19%) 5 (23.8%)	32 (43.8%) 7 (9.6%) 15 (20.5%) 19 (26%)	0.317
**Prosocial scale**				
Average Low Slightly low Very low	23 (69.7%) 2 (6.1%) 3 (9.1%) 5 (15.2%)	16 (76.2%) 2 (9.5%) 1 (4.8%) 2 (9.5%)	63 (86.3%) 2 (2.7%) 3 (4.1%) 5 (6.8%)	0.506

- Chi square test / Fisher Exact test was used to compare qualitative data between two groups

*: Significant difference (p value ≤ 0.05)

#: Significant difference between moderately severe and severe

$: Significant difference between moderately severe and profound

The relation of SDQ to the different age group is shown in Table 4. On stratification of the children with HI according to the age, at applying the psychometric assessment in to (preschoolers; 4–6 years old, early childhood; 6–12 years old and late childhood; 12–18 years old), Our study found that children with HI in early childhood have more peer problems (86%) with different degrees and only (14%) of them with average score followed by children in late childhood group (75% abnormal peer relationship problem and 25% with average score) and lastly children in preschool group (47.8% abnormal peer problem and 52.2% with average score). We noticed that peer relationship problems are less evident in the preschool group as children in the preschool group do not enter the school and do not have peers; their relations are only to their family.

**Table 4:** Relation between SDQ and age group

SDQ Scale	Preschool 4–6 years *N* = 46	Early childhood 6–12 years *N* = 57	Late childhood > 12 years *N* = 24	*P* value
Emotion scale				
Average Raised High Very high	22 (47.8%) 4 (8.7%) 11 (23.9%) 9 (19.6%)	25 (43.8%) 9 (15.8%) 5 (8.8%) 18 (31.6%)	8 (33.3%) $ 9 (37.5%) 3 (12.5%) 4 (16.7%)	0.023*
**Conduct scale**				
Average Raised High Very high	23 (50%) 15 (32.6%) 4 (8.7%) 4 (8.7%)	25 (43.9%) # 8 (14%) 14 (24.6%) 10 (17.5%)	13 (54.2%) 3 (12.5%) 6 (25%) 2 (8.3%)	0.071
**Hyperactivity scale**				
Average Raised High Very high	28 (60.9%) 4 (8.7%) 7 (15.2%) 7 (15.2%)	38 (66.7%) 6 (10.5%) 6 (10.5%) 7 (12.3%)	20 (83.3%) 0 (0%) 3 (12.5%) 1 (4.2%)	0.455
**Peer relationship scale**				
Average Raised High Very high	24 (52.2%) 12 (26.1%) 4 (8.7%) 6 (13%)	8 (14%) # 19 (33.3%) 15 (26.3%) 15 (26.4%)	6 (25%) 7 (29.2%) 5 (20.8%) 6 (25%)	0.003*
**Total scale**				
Average Raised High Very high	23 (50%) 4 (8.7%) 6 (13%) 13 (28.3%)	22 (38.6%) 5 (8.8%) 12 (21.1%) 18 (31.6%)	10 (41.7%) 7 (29.2%) 2 (8.3%) 5 (20.8%)	0.125
**Prosocial scale**				
Average Low Slightly low Very low	37 (80.4%) 0 (0%) 4 (8.7%) 5 (10.9%)	43 (75.4%) 5 (8.8%) 3 (5.3%) 6 (10.5%)	22 (91.7%) 1 (4.2%) 0 (0%) 1 (4.2%)	0.255

Chi square test / Fisher Exact test was used to compare qualitative data between two groups

*: Significant difference (p value ≤ 0.05)

#: Significant difference between moderately severe and severe

$: Significant difference between moderately severe and profound

However, more emotional problems were noticed in children in the late childhood group (66.7%) with different degrees and only (33.3%) with average score followed by children in early childhood group (56.2% had abnormal score and 43.8 with average score) followed by children in preschool group (52.2% had abnormal score and 47.8% with average score). It was noticed that peer relationship problems decreased and emotional problems are more evident in children in the late childhood group as they become more somatic, anxious, depressed, clingy, and fearful. No significant differences were found between the three age groups regarding other SDQ scales, including conduct scale, hyperactive scale, total scale, and prosocial scale.

Table 5 demonstrates the relation between SDQ and the type of hearing device used. There were no significant differences between the HA group and the CI group as regards emotional, conduct, hyperactivity, peer relationship, total, and prosocial scale.

**Table 5:** Relation between SDQ and type of hearing device used

SDQ scale	HA group *N* = 71	CI group *N* = 56	*P* value
Emotion scale			
Average Raised High Very high	29 (40.8%) 13 (18.3%) 9 (12.7%) 20 (28.2%)	26 (46.4%) 9 (16.1%) 10 (17.9%) 11 (19.6%)	0.613
**Conduct scale**			
Average Raised High Very high	(47.9%)34 10 (14.1%) 15 (12.1%) 12 (16.9%)	27 (48.2%) 16 (28.6%) 9 (16.1%) 4 (7.1%)	0.112
**Hyperactivity scale**			
Average Raised High Very high	50 (70.4%) 5 (7%) 7 (9.9%) 9 (12.7%)	36 (64.3%) 5 (8.9%) 9 (16.1%) 6 (10.7%)	0.711
**Peer relationship scale**			
Average Raised High Very high	18 (25.4%) 19 (26.8%) 18 (22.4%) 16 (22.5%)	20 (35.7%) 19 (33.9%) 6 (10.7%) 11 (19.6%)	0.149
**Total scale**			
Average Raised High Very high	31 (43.7%) 10 (14.1%) 9 (12.7%) 21 (29.6%)	24 (42.9%) 6 (10.7%) 11 (19.6%) 15 (26.8%)	0.720
**Prosocial scale**			
Average Low Slightly low Very low	54 (76.1%) 4 (5.6%) 5 (7%) 8 (11.3%)	48 (85.7%) 2 (3.6%) 2 (3.6%) 4 (7.1%)	0.673

- Chi square test / Fisher Exact test was used to compare qualitative data between two groups

*: Significant difference (p value ≤ 0.05)

Table 6 shows no significant difference was found in the SDQ regarding the side of amplification, either bilateral, right or left. However, we noticed that the children who received bilateral hearing amplification performed clinically better than those with unilateral amplification; however, the difference was still statistically insignificant.

**Table 6:** Relation of SDQ to site of used amplification (monaural Vs binaural )

SDQ Scale	Bilateral *N* = 45	Right *N* = 62	Left *N* = 20	*P* value
Emotion scale				
Average Raised High Very high	24 (53.3%) 5 (11.1%) 8 (17.8%) 8 (17.8%)	25 (40.3%) 13 (1%) 7 (11.3%) 17 (27.4%)	6 (30%) 1 (5%) 7 (35%) 6 (30%)	0.075
**Conduct scale**				
Average Raised High Very high	25 (55.6%) 9 (20%) 5 (11.1%) 6 (13.3%)	28 (45.2%) 8 (12.9%) 19 (30.6%) 7 (11.3%)	8 (40%) 7 (35%) 2 (10%) 3 (15%)	0.083
**Hyperactivity scale**				
Average Raised High Very high	36 (80%) 3 (6.7%) 1 (2.2%) 5 (11.1%)	37 (59.7%) 11 (17.7%) 5 (8.1%) 9 (14.5%)	13 (65%) 2 (10%) 4 (20%) 1 (5%)	0.087
**Peer relationship scale**				
Average Raised High Very high	15 (33.3%) 10 (22.2%) 12 (26.7%) 8 (17.8%)	20 (32.3%) 7 (11.3%) 21 (33.9%) 14 (22.6%)	3 (15%) 7 (35%) 5 (25%) 5 (25%)	0.256
**Total scale**				
Average Raised High Very high	25 (55.6%) 8 (17.8%) 3 (6.7%) 9 (20%)	25 (40.3%) 6 (9.7%) 10 (16.1%) 21 (33.9%)	5 (25%) 6 (30%) 3 (15%) 6 (30%)	0.084
**Prosocial scale**				
Average Low Slightly low Very low	36 (36%) 2 (4.4%) 2 (4.4%) 5 (11.1%)	47 (75.8%) 4 (6.5%) 4 (6.5%) 7 (11.3%)	19 (95%) 0 (0%) 1 (5%) 0 (0%)	0.611

- Chi square test / Fisher Exact test was used to compare qualitative data between two groups

*: Significant difference (p value ≤ 0.05)

As shown in Table 7, the conduct and hyperactivity scale of SDQ was mostly affected by the female sex, followed by age at diagnosis for the conduct scale and degree of discrimination for the hyperactivity scale. However, the total scale of SDQ was mostly affected by age at diagnosis and degree of discrimination. R square range from (0.092–0.168) for SDQ scales, this indicates that these factors (age, age at diagnosis, female sex, degree of hearing loss, school achievement, degree of discrimination, delay time of intervention, laterality and aiding device responsible for 9–17% of the total variance of SDQ scales.

**Table 7:** Multiple linear regression analysis for factors affecting scales of SDQ

Independent variable	Emotional scale		Conduct scale		Hyperactivity scale		Peer relationship scale		Total scale		Prosocial scale	
	Adjusted B#	*P* value	Adjusted B#	*P* value	Adjusted B#	*P* value	Adjusted B#	*P* value	Adjusted B#	*P* value	Adjusted B#	*P* value
Age	-0.032	0.727	-0.080	0.230	-0.142	0.146	0.020	0.727	-0.248	0.299	0.065	0.322
Age at diagnosis	0.101	0.392	0.180	0.034*	0.213	0.089	0.117	0.113	0.606	0.047*	-0.151	0.074
Sex (Female)	0.260	0.590	-0.790	0.024*	-1.15	0.025*	0.109	0.717	-1.752	0.162	0.483	0.164
Degree of hearing loss	1.224	0.195	0.429	0.525	1.067	0.286	0.713	0.226	3.38	0.166	0.119	0.860
School achievement	0.415	0.351	-0.371	0.245	-0.416	0.378	0.382	0.170	0.104	0.928	0.267	0.402
Degree of discrimination	0.395	0.095	0.325	0.055	0.515	0.040*	0.082	0.574	1.335	0.030*	-0.146	0.388
Delay time of intervention	-0.266	0.160	-0.189	0.165	-0.066	0.742	0.008	0.943	-0.577	0.238	-0.112	0.409
Laterality (bilateral)	1.196	0.078	0.395	0.415	0.618	0.388	0.298	0.479	2.321	0.185	0.736	0.130
Aiding device (CI)	-0.227	0.774	0.580	0.308	1.090	0.195	-0.136	0.783	1.548	0.450	-0.013	0.982

# Adjustment was made for age, age at diagnosis, female sex, degree of hearing loss, school achievement, degree of discrimination, the delay time of intervention, laterality, and aiding device

*: Significant difference (p-value ≤ 0.05)

## Discussion

Our study involved 127 hearing-impaired children, aged 4 to 17 years, who were receiving hearing amplification and following up at the audiovestibular unit in the ENT department. These children were referred to the child psychiatry clinic in the pediatrics department, where the Strengths and Difficulties Questionnaire (SDQ) was administered by an expert psychologist. The SDQ results were analyzed in relation to the degree of hearing loss, the child’s age, and the type and side of the amplification used to assess the impact of these factors on the SDQ score.

General speaking our results of SDQ scores in children with HI are much higher than those for healthy children as shown by a study conducted in Egypt to determine the prevalence of emotional and behavioral problems among 476 normal adolescent school children aged 13–17 years, the total difficulty scores and five subscales scores were considerably lower than in our study. In contrast to our study, in which the total scale score ranges from above 50% to above 75%, they found that 18.5% of normal adolescent school students had abnormal behavior, with the highest proportion of abnormal behavior was for emotional problems followed by conduct problems, hyperactivity/inattention problems, and lastly, peer relationship problems [40]. However, in our study the highest proportion of abnormal behavior differ according to the stratification of the patients according to the degree of hearing loss, age at presentation, type of hearing aids and side of amplification.

### I-Effect of degree of HL

On stratifying the patients into three groups according to the severity of hearing loss-moderately severe, severe, and profound hearing loss, our results revealed no significant differences in SDQ scale outcomes between the three groups, except for conduct behavior. Conduct Problems were more evident in children with moderately severe hearing loss. More than half of the children with moderately severe hearing loss (60.6%) had conduct problems with different degrees [About 30.3% were in the very high score (i.e., had very high conduct problems)]. In comparison, children with severe and profound hearing loss groups had conduct problems in 52.4% and 48% respectively with different degrees [only 9.5% and 5.5% were in the very high score].

These findings contrast with other research, which found that the severity of conduct behavior is directly proportional to the severity of hearing loss [8, 22, 28, 41]. Those studies attributed this relationship to the fact that the greater HI in childhood leads to more pronounced delays and deficits in the acquisition of spoken and written language, which have been postulated to negatively impact behavior [22, 42].

However, our findings can be explained by the fact that children with the least severe hearing loss in our study exhibited the highest levels of conduct behavior for the following reasons: ***First explanation***, children with moderately severe hearing loss experienced a greater delay in diagnosis and intervention (3 years for diagnosis and 1 year for intervention) compared to those with severe hearing loss (3 years for diagnosis and 0.5 years for intervention) and profound hearing loss (1.5 years for both diagnosis and intervention). This delay in diagnosis and intervention may be attributed to various psychosocial factors, such as parental denial and initial resistance before accepting their child’s hearing loss and the need for HA. Since these children appeared to respond to sounds, parents might have assumed that HA were unnecessary or feared the social stigma associated with their use [43].

This delay in diagnosis and interference causes more delay in speech and language development, which in turn has a greater negative impact on behavior of the hearing impaired children [42]. The central auditory system may degenerate as a consequence of delayed problem management, resulting in lost opportunities for education and employment and a reduced quality of life [44]. It was reported that children with SNHL who receive early intervention exhibit behavior similar to that of their normal hearing (NH) peers [45].

***The second explanation***, based on our study, is that children with moderately severe hearing loss have a higher prevalence of a positive family history of hearing loss (48.5%) compared to those with severe (38.1%) and profound hearing loss (34.2%), which explains why these children had higher conduct or behavioral problems. Children with HI exhibit more indirect and physical violence when a family member has hearing loss. According to reports, one of the primary elements associated with violence is family. The presence of a family member with a disability is clearly going to have a direct impact on the behaviors of children with and without HI [46].

### II- Effect of age of children

On stratification of the children with HI according to the age into (preschoolers; 4–6 years old, early childhood; 6–12 years old and late childhood; 12–18 years old), our study found that children in early childhood period had significantly more peer relationship problems (86%) with different degrees followed by children in late childhood group (75% abnormal peer relationship) and lastly children in preschool group (47.8% abnormal peer relationship). The lower incidence of peer relationship in preschoolers may be attributed to their limited social interactions, as their relationships are primarily confined to family members and relatives. Most have not yet started school or developed friendships outside the family.

Stevenson et al. reported that peer relationship difficulties are the most common problem among children with reduced hearing [23]. Children with HI are twice as likely to be bullied by peers compared to those with normal development [47, 48]. Even mild hearing loss can cause children to miss important social signs during play, making them more vulnerable to bullying. As a result, they often experience feelings of shame or inequality [46].

In our study, we also observed that peer relationship problems decreased in the late childhood group, while emotional disturbances significantly increased in this age group compared to the younger two groups. The late childhood group showed a higher prevalence of emotional problems (66.7%) with varying degrees of severity compared to the early childhood group (56.2% abnormal scores) and the preschool group (52.2% abnormal scores). This suggests that children in late childhood tend to exhibit more somatic, anxious, depressed, clingy, and fearful characteristics.

It is known that verbal and psychological bullying is common as age increases, but physical bullying decreases [49, 50]. Thus, we postulated that; the untreated peer problems in the younger age led to more emotional disturbances in late childhood and adolescence, and this finding was evidenced by one study showed that; peer bullying creates negative effects such as anxiety, low self-esteem, and depression which preventing victims from integrating into the school environment [51].

Furthermore, there is a link between HI and moderate to severe depression, and it is a major source of anxiety in children and adolescents [8, 52]. It has been discovered that children with HI, particularly SNHL, will become socially isolated and find it challenging to participate fully in activities with their families, friends, neighbors, and even at school or work. Children with HI will have feelings of inferiority, irritability and anger, anxiety, introversion, and fear. As a result of this scenario, it will hinder their development of affection, emotions, and conduct. They ultimately have less developed personalities and social skills compared to their peers [16, 53, 54].

On comparing the prevalence of emotional and behavioral problems with adolescents having a specific learning disability, apart from hyperactivity inattention behaviors and prosocial problems, our study revealed a higher prevalence of emotional and behavioral problems among hearing-impaired children than those with specific learning disability [55]. This is consistent with past studies showing that children and adolescents with intellectual disabilities may not be able to exhibit some types of behavior because they require more sophisticated social-cognitive abilities (like perspective-taking) [56].

On comparing the prevalence of emotional and behavioral problems with adolescents having other health conditions like underlying heart problems, apart from prosocial symptoms, our study showed higher scores in total and other behavioral problems [57]. That may indicate that children with underlying heart conditions may exhibit fewer positive social behaviors compared to norms.

### III- Effect of type of used amplification (HA vs CI)

On stratifying the patients according to the used hearing amplification (HA vs. CI), our results revealed that there is no significant difference observed in the 5 scales of the SDQ despite the more severe hearing loss in CI group (98.2% had profound hearing loss) than the other HA group (only 25.4% had profound hearing loss). The explanation of our findings may be attributed **first** to the age of diagnosis and the shorter delay to interference in children using CI compared to those using HA. Health insurance policies for CI often require early diagnosis and implantation before the age of five, which coincides with the critical period for speech development. Additionally, children who receive CI benefit from extra counseling as part of their rehabilitation services. As a result, these children are more likely to develop oral language skills, integrate into regular schools, and engage in social activities [58].

**The second explanation is** that less than half of children (46.5%) who use HA in our study had moderate degree hearing loss, 28.2% of severe degree hearing loss. It is known that most children with a milder degree of hearing loss refuse to wear the HA due to concerns about stigmatization or discrimination. They also fear being bullied by peers and even relatives, which leads to irregular use of the devices [43]. This, in turn, results in a greater difficulty in communication and forming interpersonal relationships [59]. The more avoidance of environmental interaction, the lower their self-esteem becomes, and the stronger their sense of social neglect, ultimately contributing to increased behavioral problems [60, 61].

### IV-Effect of site of used hearing amplification (Rt, Lt or bilateral )

Our results revealed no significant difference was found in the SDQ scales as regards to the side of used hearing amplification, right, left or bilateral. However, we noticed that the children who received bilateral hearing amplification performed clinically better than those with unilateral amplification, although the difference remained statistically insignificant.

It is well established that one hemisphere is of greater influence on brain functions [62]. Left hemisphere dominance is observed in 95–98% of right-handed individuals and in 70–80% of left-handed, NH subjects for speech perception and production [63–65]. On the other hand, most of these participants exhibit right hemisphere dominance for prosodic language functions, such as accentuation and intonation. While the left hemisphere is dominant for speech and language processing in most people [66], language functions are primarily lateralized to the left hemisphere, whereas emotions particularly negative ones such as arousal and attention—are more commonly lateralized to the right hemisphere [67]. The lateralization of brain function allows each hemisphere to specialize in different mental tasks [68].

Both ears provide auditory input to the auditory cortex, as was recently discovered [69–71]. The degree of asymmetry between the two hemispheres is believed to vary depending on the specific mental function, and it is believed to be asymmetrically involved in many attentional, cognitive, and emotional functions [72]. This may explain our results.

Additionally, a study discovered that when unilateral reafferentation of the left ear occurs during a crucial early stage of life when the brain is (still) plastic, it can cause a reorganization of language skills in the right hemisphere [73]. The first 3.5 years of life are considered the most plastic period for the brain [74, 75]. As a result, patterns of left hemisphere dominance are maintained when the right ear is amplified or when the left ear is amplified later in life [66]. Parallel to studies conducted among kids with typical hearing, simultaneous and bilateral hearing augmentation in children produces notable improvements in understanding spoken words through the right ear. It may contribute to the appropriate development of central auditory pathways and be crucial for the maturation of communication and language [76].

On determining the confounding variable to develop emotional and/or behavioral problems, our study found that increased age at diagnosis is the main confounding variable for increased total scale score of behavioral problems and specifically conduct behavior score. This agrees with other literature studies that have also shown that early detection of hearing loss was associated with favorable lower incidence of behavioral and emotional problems [42, 77, 78].

Surprisingly, our study found that, increased degree of speech discrimination is significantly associated with increased total scale score and specifically the hyperactivity inattention scores. Lewis et al. also found that, moderate–severe HI group demonstrated more symptoms of inattention and hyperactivity/impulsivity than the children with severe hearing loss and, they attributed the condition to the higher scores to language impairment in these children [79].

However, our study found that the female gender has a significantly lower risk for developing conduct and hyperactivity inattention behaviors rather than male children with HI. This aligns with the findings of Theunissen et al., who discovered that being male was significantly associated with higher levels of delinquency and attention deficit hyperactivity disorder [8].

## Conclusions

Our study found that children with HI who received hearing amplifications still exhibited emotional and behavioral problems, which were largely influenced by the timing of diagnosis and age of intervention. Specifically, children diagnosed with moderately severe hearing loss showed significantly more conduct issues compared to those with severe or profound hearing loss, likely due to having a later diagnosis and intervention. Additionally, children in the primary school age group had significantly more peer-related problems than preschoolers or adolescents. However, as they aged, children in late childhood experienced fewer peer relationship problems but developed more significant internalizing issues, such as anxiety, depression, and clingy or fearful behaviors.

Despite the fact that children using CI had profound hearing loss, no significant difference was found between this group and HA group in emotional or behavioral problems, which emphasizes the fact that early diagnosis and interventions are the main predictors of the psychosocial outcome in children with HI. Regarding the side of amplification, whether right, left, or bilateral, no significant difference was found in the children’s emotions or behaviors. This suggests that early intervention in the neuroplastic brain may facilitate the reorganization of language functions. Therefore, the timing of hearing amplification seems to play a more important role in influencing emotions and behaviors than the side of amplification.

These problems need to be scanned early, detected, and treated thoroughly in children with HI. Additionally, there is a need for greater community support and psychological assistance for both the child and their family, particularly when the child reaches school age. Emotional disturbances should also be screened and managed promptly during adolescence. Finally, regular follow-ups and psychiatric support are essential to help children cope with the psychological challenges that may arise beyond their HI.

### Limitations

The cross-sectional design of our study carries the limitation in the determination of the reasoning of the associations between findings. Also, the response bias by the parents of the children rather the observational methods of behavioral assessment carry another limitation in this study as subjective questionnaire results cause potential biases. The small sample size may limit generalization of the results and further researches with larger sample size are recommended to support our results, however our study may raise attention to expect the presence of any behavioral and emotional problems in children and adolescent with HI according to their degree of hearing loss and age of the child and to refer and perform more specific psychological tests accordingly to help and support those children.

## Data Availability

The data that support the findings of this study are not openly available due to reasons of sensitivity and are available from the corresponding author upon reasonable request.

## Abbreviations

HI: Hearing impairment
ABG: Air bone gap
CI: Cochlear implant
CHL: Conductive hearing loss
HA: Hearing aids
NH: Normal hearing
SD score: speech discrimination score
SDQ: Strengths difficulty questionnaire
SNHL: Sensorineural hearing loss
